# Impact of Cortisol-Cosecretion on Adrenal Venous Sampling Results in Primary Aldosteronism: Study of 225 Cases

**DOI:** 10.3390/biomedicines12112430

**Published:** 2024-10-23

**Authors:** Cristina Lamas, Marta Araujo-Castro, Lukas Ostermair, Erik Petersenn, Paola Parra Ramírez, Ángel Rebollo-Román, Isabel Stuefchen, Denise Bruedgam, Jorge Gabriel Ruiz-Sanchez, Theodora Michalopoulou, Carolina M. Perdomo, Felicia A. Hanzu, Christian Adolf, Martin Reincke

**Affiliations:** 1 Endocrinology and Nutrition Department, Complejo Hospitalario Universitario de Albacete, 02001 Albacete, Spain; 2 Endocrinology Department, Hospital Ramón y Cajal, 28034 Madrid, Spain; 3 Medizinische Klinik und Poliklinik IV, Ludwig-Maximilians-Universität München, 81377 Munchen, Germany; lukas.ostermair@med.uni-muenchen.de (L.O.); erik.petersenn@med.uni-muenchen.de (E.P.); isabel.stuefchen@med.uni-muenchen.de (I.S.); denise.bruedgam@med.uni-muenchen.de (D.B.); christian.adolf@med.uni-muenchen.de (C.A.); martin.reincke@med.uni-muenchen.de (M.R.); 4 Endocrinology and Nutrition Department, Hospital Universitario La Paz, 28046 Madrid, Spain; paola.parra@salud.madrid.org; 5 Endocrinology and Nutrition Department, Hospital Reina Sofía, 14004 Córdoba, Spain; 6 Endocrinology and Nutrition Department, Hospital Fundación Jiménez Díaz Madrid, 28040 Madrid, Spain; gajo_saru@hotmail.com; 7 Endocrinology and Nutrition Department, Hospital Universitari de Tarragona Joan XXIII, 43005 Tarragona, Spain; tmichalopoulou.hj23.ics@gencat.cat; 8 Endocrinology and Nutrition Department, Clínica Universidad de Navarra, 31008 Pamplona, Spain; cperdomo@unav.es; 9 Endocrinology and Nutrition Department, Hospital Clinic de Barcelona, 08036 Barcelona, Spain; fhanzu@clinic.cat

**Keywords:** primary aldosteronism, autonomous cortisol secretion, dexamethasone suppression test, adrenal venous sampling

## Abstract

**Background/Objectives**: Mild autonomous cortisol secretion (MACS) can coexist with primary aldosteronism (PA). The purpose of our study was to evaluate whether (MACS) influences parameters analyzed during adrenal venous sampling (AVS) in patients with PA. **Methods**: Patients with PA from the SPAIN-ALDO Registry and the German Conn’s Registry with available 1 mg-dexamethasone suppression test (DST) and AVS were included. MACS was defined as a post-DST cortisol > 1.8 µg/dL in the absence of specific signs and symptoms of Cushing’s syndrome. **Results**: Two-hundred and twenty-five patients were included, 98 (43.6%) of whom had concomitant MACS. The mean age was 54 ± 10 years and 37.3% were women. AVS was performed by simultaneous catheterization of both adrenal veins and analysis of basal samples in 157 patients (69.8%), with both basal and post-ACTH samples in 15 patients (6.7%), and during continuous ACTH infusion in 53 patients (23.6%). AVS was considered technically unsuccessful in 40 cases (17.8%), suggesting unilateral secretion in 106 (47.1%) and bilateral secretion in 79 (35.1%). We did not find significant differences in the percentage of unilateral and bilateral results, cortisol, corrected aldosterone, or selectivity indices in the dominant and non-dominant veins, nor in the lateralization index or the contralateral suppression index between patients with and without MACS. They also had similar rates of surgical treatment and biochemical and clinical response. **Conclusions**: Although pathophysiological reasoning suggests that MACS could hinder AVS identification of unilateral forms of PA, our data suggest that such interference, if it exists, is of moderate clinical relevance.

## 1. Introduction

Primary aldosteronism (PA) is the most common cause of secondary hypertension [1]. Compared to patients with essential hypertension, patients present with more severe myocardial fibrosis, left ventricular hypertrophy, and vascular remodeling, a process that involves the hypertrophy of vascular smooth muscle cells, increased production of extracellular matrix components and inflammation, leading to stiffer and less compliant blood vessels and increased risk of cardiovascular events. Higher rates of atrial fibrillation, heart failure, and cardiovascular and cerebrovascular diseases are known complications of PA [2,3,4].

Some cases of PA are due to hypersecretion of aldosterone predominantly by a unilateral source, usually by an aldosterone-producing adenoma, and more rarely by asymmetric hyperplasia. These lateralized cases can be cured or significantly improved by adrenalectomy. On the contrary, patients with bilateral aldosterone hypersecretion cannot expect a surgical cure and must be managed with medical treatment, preferably mineralocorticoid receptor antagonists. Good discrimination between unilateral and bilateral forms of PA is therefore essential [1,5]. Clinical, biochemical, and radiological parameters are useful [6], but adrenal vein sampling (AVS) is considered the gold standard for this distinction. It analyzes the aldosterone concentration in both adrenal veins and in the inferior vena cava or a peripheral vein, comparing them with each other to identify the origin of the hypersecretion.

To avoid the effect of blood dilution, aldosterone levels are corrected by relating them to cortisol concentrations in the same blood sample. Cortisol levels in venous samples are also used to confirm the correct positioning of catheters in the adrenal veins [7,8].

Recently, mild autonomous cortisol secretion (MACS) coexisting with PA has been described [9,10,11,12,13,14,15]. In fact, these cases are not rare, reaching prevalence rates of up to 30% of PA cases according to some studies. For example, an analysis of the patients included in the Spanish registry of primary hyperaldosteronism (SPAIN-ALDO) showed that 51 of 176 patients (29%) had a cortisol; after a nocturnal 1 mg-dexamethasone suppression test (DST), greater than 1.8 µg/dL [16]. This percentage was 21% in a Japanese study [10] and 23% in a recent Korean study [14]. When interpreting the data from AVS, the question arises as to whether MACS can modify the parameters that are usually evaluated in the test. Goupil et al. demonstrated that the presence of a cortisol-producing adenoma led to low selectivity indices in the adrenal vein contralateral to the adenoma and falsely low aldosterone/cortisol ratios in the ipsilateral vein [17], but this has not been proven in cases of MACS (without Cushing’s syndrome). Other authors have explored the interference of MACS in the interpretation of AVS data, but the results have been contradictory [18,19].

The objective of our study was to evaluate in depth, in a large cohort of patients with PA, whether MACS modifies the parameters that are analyzed in AVS and the interpretation of the test, so that it could lead to errors in the therapeutic approach (operating on patients with bilateral secretion or not operating on patients with unilateral secretion).

## 2. Materials and Methods

### 2.1. Patients and Definitions

Adult patients with confirmed PA according to the Endocrine Society Practice Guideline [1] who had undergone AVS and an overnight 1 mg DST were included. Patients who presented with clinical signs of overt Cushing’s syndrome or were receiving chronic glucocorticoid treatment were excluded. Demographics, clinical, biochemical, radiological, and follow-up data were extracted from the medical records and recorded in the SPAIN-ALDO Registry or the German Conn’s Registry in a pseudonymized manner using an identification number. SPAIN-ALDO is a RedCap database approved by the Research Ethics Committee of Ramón y Cajal Hospital in Madrid. The German Conn’s Registry is a RedCap database approved by the Ethics Committee of the Medical Faculty of the Ludwig-Maximilians Universität Munich. Sixty-six per cent of the German cohort were included in a previous study [19].

PA was confirmed by a saline infusion test in 151 (75.1%) cases, by a captopril challenge test in 47 (23.4%), and another test in 3 (1.5%). Twenty-four patients (10.7%) did not undergo confirmation testing because of spontaneous hypokalemia, plasma renin below detection levels, and plasma aldosterone concentration (PAC) > 20 ng/dL [1]. Adrenal imaging was performed using computed tomography (CT) in 130 patients (57.8%), magnetic resonance imaging (MRI) in 56 (24.9%), and both CT and MRI in 32 patients (14.2%). No adrenal imaging was registered in 7 patients (3.1%). Patients with normal adrenals were excluded for the calculation of the adenoma size statistics. Overnight DST was performed by administering 1 mg of dexamethasone at 11:00 p.m. and analyzing the cortisol concentration in a blood sample drawn at 8:00 a.m. the next morning. MACS was defined as a post-DST cortisol > 1.8 µg/dL in the absence of specific signs and symptoms of Cushing’s syndrome [20].

AVS was performed by simultaneous catheterization of both adrenal veins under basal conditions without ACTH stimulation in 157 patients (69.8%), before and after a bolus of 250 µg of ACTH in 15 patients (6.7%), and during continuous infusion of ACTH at 50 µg/h in 53 patients (23.6%). Correct catheterization of the adrenal veins was established using the selectivity index (the ratio between the cortisol concentration in the adrenal vein and that in the inferior vena cava or a peripheral vein), which had to be higher than 2 when performed without ACTH and higher than 4 after ACTH administration. The diagnosis of unilateral PA was established by the treating physician, mostly but not solely based on a lateralization index (the ratio between the cortisol-corrected aldosterone in the dominant vein and that of the non-dominant vein) > 4. We also calculated the contralateral suppression index (the ratio of the cortisol-corrected aldosterone of the non-dominant vein with respect to the cortisol-corrected aldosterone of the inferior vena cava or a peripheral vein), which can be used as a surrogate marker of unilateral secretion when the lateralization index does not reach the established cut-off. All analyses were routinely performed in the respective laboratories of the participating centers.

The definitions of biochemical and clinical cure for PA after adrenalectomy were based on the Primary Aldosteronism Surgical Outcome (PASO) classification system [21].

### 2.2. Statistical Analysis

For statistical analysis, categorical variables were expressed as frequency counts and percentages. Continuous variables were summarized as the mean and standard deviation when the normality assumption was fulfilled, and as the median and interquartile range (IR) when normal distribution was not confirmed. The Shapiro–Wilk test was used to assess normality. Chi-square and Fisher’s exact tests were used to compare proportions. When comparing two numeric samples, we used Student’s t or Wilcoxon–Mann–Whitney depending on normality, which was also the case when more than two samples were compared, and we had to choose between ANOVA and the Kruskal–Wallis test. All hypotheses were tested for differences (two tails) using an alpha of 0.05 as the statistical threshold. All statistical analyses were performed using R software (v: 4.3.2).

## 3. Results

### 3.1. Baseline Characteristics of the Cohort

One hundred and twenty patients from the German Conn’s Registry and 105 patients from the SPAIN-ALDO Registry, diagnosed between 2012 and 2023, met the inclusion criteria and were included in the study. The mean age was 54 ± 10 years and 37.3% were women. All patients, except one, had arterial hypertension, diagnosed at the age of 42 ± 11 years, and 38.2% needed three or more antihypertensive drugs at diagnosis. Hypokalemia was observed in 134 patients (59.6%). In the imaging tests, 132 patients (63.4%) had unilateral adrenal adenomas, 23 (11.1%) had bilateral adrenal adenomas, and 53 (25.5%) had normal-looking adrenal glands. The mean maximum diameter of the dominant nodule was 17 mm (13–20.5).

When comparing patients in the Spanish registry with those in the German registry, we found some differences, which are summarized in Table 1, and come down to a higher rate of MACS (despite a non-significant difference in post-DST cortisol), milder aldosteronism, and better performance of AVS in the German cohort (Table 1).

One hundred and twenty-seven patients (56.4%) had a cortisol value after DST ≤ 1.8 µg/dL and 98 patients (43.6%) > 1.8 µg/dL. We did not find differences in most of the demographic and clinical parameters between these two groups of patients, (Table 2), except for a smaller adenoma size in patients with DST ≤ 1.8 µg/dL (16 (11–20) vs. 18 (15–28) mm, *p* = 0.0027). The demographic, clinical, biochemical, and radiological parameters at the time of diagnosis for the entire cohort and for each category of cortisol autonomy are displayed in Table 2.

### 3.2. Adrenal Vein Sampling Results

AVS was considered unsuccessful in 40 cases (17.8%, due to unsuccessful catheterization of the right adrenal vein in most cases), indicative of unilateral secretion in 106 (47.1%) and indicative of bilateral secretion in 79 (35.1%). Procedures performed without ACTH had better performance than procedures performed with ACTH bolus or ACTH infusion (5.1%, 46.7%, and 47.2% unsuccessful procedures, respectively; *p* > 0.001), probably due to the better performance of the technique in the German cohort, with all the samplings performed without ACTH. The coexistence of MACS did not lead to different success rates in AVS with ACTH administration. For AVS without ACTH, the success rate was even better in patients with MACS, although the number of patients with unsuccessful AVS was small (n = 8) (Table 3).

**Figure 1:**
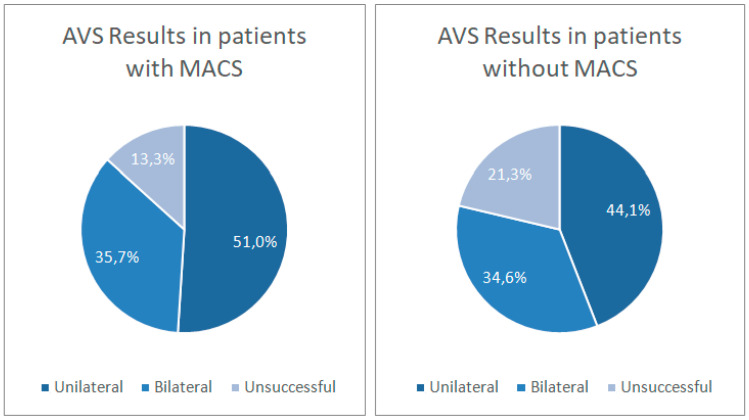
AVS Results in patients with and without MACS (*p* = 0.277 for comparison).

We did not find differences between patients with and without MACS in the percentage of unilateral and bilateral results (51.0% and 35.7% for patients with MACS and 44.1% and 34.6% for patients without MACS, *p* = 0.2767; Figure 1), nor in cortisol concentration in the dominant and non-dominant veins, dominant and non-dominant corrected aldosterone, dominant and non-dominant selectivity indices, lateralization index, or contralateral suppression index in basal and ACTH-stimulated samples (Table 4). All these comparisons were repeated by comparing the subgroup of 18 patients (8%) with DST > 5 µg/dL with those with DST ≤ 5 µg/dL, with similar results.

### 3.3. Surgical Outcomes

Ninety-one patients (40.4%) underwent surgery: their AVS results were unilateral secretion in 67 (73.6%), bilateral secretion in 8 (8.8%), and unsuccessful sampling in 16 (17.6%). Patients with MACS (n = 43) had a similar probability of being operated on as patients without MACS (n = 48) (43.9% vs 37.8%, *p* = 0.4326). This was true for every AVS result: 67.9% with MACS vs. 58% without MACS in unilateral secretion (*p* = 0.396), 4.5% vs. 17.1% in bilateral secretion (*p* = 0.1293), and 29.6% vs. 61.5% in unsuccessful AVS (*p* = 0.113).

A total of 70 out of 88 patients (79.5%) achieved a clinical response (complete or partial), and 68 of 73 (93.2%) achieved a complete biochemical response. We did not find differences in the surgical outcomes between patients with and without MACS (clinical response 77.8% vs. 81.4%, *p* = 0.2774; biochemical response 93.5% vs. 92.6%, *p* = 1.

## 4. Discussion

Since the concentration of cortisol in the adrenal veins is used in AVS both to check the correct placement of the catheters and to normalize the concentration of aldosterone, there is concern among the scientific community about how MACS can influence the interpretation of AVS results. It is reasonable to think that, in cases of unilateral cortisol secretion, coming from the same adenoma as aldosterone (or at least the same adrenal gland), the aldosterone/cortisol ratio could be reduced on that side and increased on the contralateral side, which would minimize the lateralization index and prevent the correct identification of unilateral ipsilateral aldosterone secretion. However, we did not find worse AVS success rates in patients with MACS; on the contrary, they had a significantly higher success rate when AVS performed without ACTH was independently analyzed. For the patients with ACTH-stimulated AVS, patients with MACS had less successful procedures (38.5% vs. 51.2%) but the difference was not significant, probably due to the low number of patients in the MACS group (n = 23, *p* = 0.264, statistical power 0.4), all coming from the Spanish cohort. Moreover, our data did not show differences in the lateralization ratios or in the interpretation of the test results between patients with and without MACS.

A possible explanation for this is that the magnitude of autonomous cortisol secretion is not large enough to significantly modify AVS indices. In fact, in a study by Heinrich et al. [19], a lower lateralization index was found, but only for the few patients with unilateral secretion and DST > 5 µg/dL, a finding that was not confirmed in our series. Another possible explanation is that we are incorrectly assuming that autonomous cortisol and aldosterone secretion always originate from the same gland, which may be true in some patients, but not in others. Just as autonomous aldosterone secretion can be unilateral or bilateral, the same can occur with cortisol, which can originate from a unilateral adenoma, but also from bilateral adenomas or bilateral hyperplasia, further complicating the interpretation of the data. Distinguishing unilateral from bilateral cortisol secretion is difficult, especially in the case of bilateral adrenal masses [23], but we do have data on the origin of aldosterone secretion in patients with MACS, which was unilateral, based on AVS, only in 50% of our patients, 75% in the series of Araujo, 42% in the series of Katabami et al., 42.9% in the series of O’Toole et al., and 57.7% in the series of Heinrich et al. [15,16,18,19].

Our unexpected negative results could also be due to an unusually high rate of false positive results in the DST test. The high prevalence of MACS in our series (43%) may support this hypothesis. False positives of DST can occur in relation to aging, interfering drugs, kidney failure, hemodilution, psychiatric diseases, or with increased serum total cortisol in the presence of high serum protein levels, as occurs under estrogen therapy [24]. They can also be the consequence of unexpectedly lor serum concentration of dexamethasone, due to malabsorption or increased clearing [25]. None of these were suspected by the attending physicians. However, our study did not include other parameters, such as ACTH, DHEA-S or free urinary or salivary cortisol in the definition of MACS, and we stuck to the most common and recommended criterion (DST > 1.8 µg/dL) [20].

When reviewing other similar studies, the results were not very different from ours. O’Toole et al. [18] analyzed AVS performed under ACTH stimulation and compared patients with and without MACS (defined by a DST > 1.8 µg/dL), and found no statistically significant differences in selectivity indices, adrenal vein cannulation rate, lateralization index, or unilateral diagnoses: however, they did find a higher concentration of cortisol in the inferior vena cava in patients with MACS. A possible limitation of their study was that it included only 21 patients with MACS [18].

Heinrich et al. [19], on the other hand, evaluated AVS performed under basal conditions without ACTH and found no statistically significant differences in the AVS success rate or the percentage of patients classified as unilateral or bilateral depending on the co-secretion of cortisol. However, they observed a tendency toward a lower lateralization index in cases with DST > 5 µg /dL (only significant for unilateral cases), with no differences between patients with DST > 1.8 µg/dL or ≤1.8 µg/dL. They also found a higher cortisol concentration in both adrenal veins, which did not translate into differences in the corrected aldosterone or selectivity, lateralization, or contralateral suppression indices. In addition, more patients with a DST > 1.8 µg/dL underwent unilateral adrenalectomy despite bilateral AVS result, with good clinical and biochemical response rates [19]. We also found more cases with surgical treatment despite a bilateral result in AVS (17.1% vs. 4.5%) or an unsuccessful AVS (61.5% vs. 29.6%) among the patients with MACS, but the difference did not reach statistical significance in our data.

Some authors have proposed metanephrine, the metanephrine-to-normetanephrine ratio, the free-to-total metanephrine ratio and adrenal androgens as better parameters than cortisol for the normalization of aldosterone in AVS [22,26,27,28,29,30], but they have not been evaluated in patients with MACS. Actually, we would expect a low utility of androgens due to their suppression in the presence of mild ACTH-independent cortisol hypersecretion. Metanephrine represents a more appealing alternative and has been evaluated with encouraging results in a single study in the presence of cortisol asymmetric secretion based on adrenal vein serum cortisol concentration [29], but cortisol suppressibility was not evaluated. More data are therefore needed to determine whether the diagnostic performance of these alternative analytes for aldosterone normalization is comparable or even superior to that of cortisol in patients with MACS. Other alternative methods are also being developed to differentiate the unilateral and bilateral forms of PA, such as 68Ga-pentixafor PET/CT or PET/MRI [31,32,33], but no prospective studies have evaluated its diagnostic accuracy in the context of MACS.

The main strength of our study is that it represents the largest series of patients with thorough information about AVS data and cortisol autonomy. It also has some limitations. The participation of multiple centers with different AVS protocols, mainly regarding ACTH administration, confers heterogeneity to the cohort, and the statistical power was substantially reduced when the analysis was carried out for homogeneous cohorts regarding the administration of ACTH. MACS diagnosis was established based on the results of a pathological DST, but information about ACTH was missing in 23.5% of the patients with MACS, so we could not completely rule out the unlikely possibility of ACTH dependency (i.e., Cushing´s disease) in every patient. The retrospective design of the study, inclusion of 35.5% of our patients in a previous study and suboptimal success rates are other limitations. 

In conclusion, although pathophysiological reasoning suggests that MACS could hinder AVS identification of unilateral forms of PA, our data and those of other authors seem to suggest that such interference, if it exists, is of moderate clinical relevance.

## Figures and Tables

**Table 1:** Differences between the German and the Spanish cohorts. Age is summarized as mean ± standard deviation, other parameters as median (interquartile range). There were no differences between both cohorts in sex, SBP, DPB, prevalence of hypokalemia, adenoma size, PAC, lateralization index or contralateral suppression index, final AVS diagnosis, percentage of patients referred to surgery or surgical outcomes. Hypertension grades are based on the ESH 2023 guidelines [22] (HT: hypertension, PAC: plasma aldosterone concentration, DRC: direct renin concentration, DST: dexamethasone suppression test, MACS: mild autonomous cortisol secretion, AVS: adrenal vein sampling, ACTH: adrenocorticotropic hormone, SBP: systolic blood pressure, DBP: diastolic blood pressure, Corrected aldosterone: aldosterone/cortisol ratio). Significant *p* values are marked in bold.

	Whole Cohort	German Cohort	Spanish Cohort	*p* Value
Age (years)	54 ± 10	52.6 ± 10.34	55.3 ± 9.4	***p*** **= 0.046**
HT grades:				***p*** **< 0.001**
1	49.8%	63.3%	32.3%	
2	29.1%	25.8%	33.3%	
3	21.1%	10.8%	34.4%	
Antihypertensive drugs				***p*** **< 0.001**
1–2	61.8%	85%	35.2%	
3	15.1%	10%	21%	
>4	23.1%	5%	43.8%	
DRC (mU/L)	2.9 (2–5.9)	3.85 (2–7.15)	1.6 (0.55–3.76)	***p*** **< 0.001**
Confirmation test				***p*** **= 0.003**
Saline infusion test	75.1%	82.5%	64.2%	
Captopril challenge test	23.4%	17.5%	32.1%	
Imaging technique				
CT	72%	51.7%	98%	***p*** **< 0.001**
MRI	39.1%	47.9%	30.4%	***p*** **= 0.007**
Adrenal imaging:				***p*** **< 0.001**
Unilateral mass	63.4%	56.2%	72.3%	
Bilateral masses	11.1%	6.1%	17%	
Normal	25.5%	37.7%	10.6%	
PostDST cortisol (µg/dL)	1.5 (0.9–2.5)	1.9 (0.8–2.8)	1.5 (1–2.1)	*p* = 0.824
MACS	43.6%	53.3%	32.4%	***p*** **= 0.003**
AVS type:				***p*** **< 0.001**
without ACTH	69.8%	100%	35.2%	
ACTH bolus	6.7%		14.3%	
ACTH infusion	23.6%		50.5%	
AVS results				***p*** **< 0.001**
Unilateral	47.1%	50%	43.8%	
Bilateral	35.1%	48.3%	20%	
Non-valid	17.8%	1.7%	36.2%	
Selectivity index				
Dominant	7.68 (3.55–28)	8.28 (3.75–33.14)	7.4 (2.31–16.6)	***p*** **= 0.020**
Non-dominant	13.8 (3.91–34.02)	18 (5.74–39.37)	5.28 (2.3–16.93)	***p*** **< 0.001**
Corrected aldosterone (ng/µg)				
Dominant	3.54(1.37–13.86)	2.18 (1.1–8.18)	10.99 (3.46–33.85)	***p*** **< 0.001**
Non-dominant	0.70 (0.32–1.50)	0.62 (0.28–1.08)	1.51 (0.67–3.29)	***p*** **< 0.001**

**Table 2:** Demographic, clinical, biochemical and radiological parameters at diagnosis in the whole cohort and in the subgroups of patients according to DST. Hypertension grades are based on the ESH 2023 guidelines [22] (DST: dexamethasone suppression test, HT: hypertension, LVH: left ventricle hypertrophy, SBP: systolic blood pressure, DBP: diastolic blood pressure, PAC: plasma aldosterone concentration, DRC: direct renin concentration; PRA: plasma renin activity, DST: dexamethasone suppression test; ACTH: adrenocorticotropic hormone, Post-test PAC: plasma aldosterone concentration after saline infusion or captopril challenge test. Significant *p* values are marked in bold.

	Whole Cohort (n = 225)	DST ≤ 1.8 µg/dL (n = 127)	DST >1.8 µg/dL (n = 98)	*p* Value
Sex (% female)	37.3%	35.4%	39.8%	*p* = 0.590
Age (years)	54 ± 10	53 ± 9	55 ± 11	*p* = 0.068
HT grades:				***p*** **= 0.045**
1	49.8%	42%	59.6%	
2	29.1%	34.4%	22.3%	
3	21.1%	23.5%	18.1%	
LVH	32.9%	36.4%	29.2%	*p* = 0.447
Antihypertensive drugs				*p* = 0.823
1–2	61.8%	61.4%	62.2%	
3	15.1%	14.2%	16.3%	
>4	23.1%	24.4%	21.4%	
Hypokaliemia	59.6%	57.5%	62.2%	*p* = 0.559
SBP (mmHg)	148 (135–160)	149 (138–160)	148 (135–164.5)	*p* = 0.792
DBP (mmHg)	91.8 (84–101)	92 (84–101)	91.5 (84–100)	*p* = 0.464
PAC (ng/dL)	20.6(15–31.2)	22.7 (15.5–31.1)	19.6 (14.3–32.4)	*p* = 0.461
DRC (mU/L)	2.9 (2–5.9)	2.3 (2–5.6)	3 (2–6.3)	*p* = 0.356
PRA (ng/mL/h)	0.29 (0.2–0.37)	0.3 (0.2–0.33)	0.25 (0.18–0.5)	*p* = 0.987
Post-test PAC (ng/dL)	14.2 (9.6–22.7)	14.9 (9.8–25)	13.7 (9.2–20.4)	*p* = 0.237
Cortisol after DST (µg/dL)	1.5 (0.9–2.5)	0.9 (0.8–1.2)	2.8 (2.1–3.9)	***p*** **< 0.001**
Adrenal imaging:				*p* = 0.088
Unilateral mass	63.4%	60.3%	67.4%	
Bilateral masses	11.1%	8.6%	14.1%	
Normal	25.5%	31%	18.5%	
Adenoma size (mm)	17 (13–21)	16 (11–20)	18 (15–28)	***p*** **= 0.003**

**Table 3:** Number of successful adrenal venous samplings depending on ACTH administration for the procedure (bolus or continuous infusion) and the coexistence of mild autonomous cortisol secretion. (DST: dexamethasone suppression test; ACTH: adrenocorticotropic hormone). Significant *p* values are marked in bold.

	DST ≤ 1.8 µg/dL	DST > 1.8 µg/dL	*p* Value
AVS without ACTH (successful/unsuccessful)	74 (90.2%)/8 (9.8%)	75 (100%)/0	***p*** **= 0.022**
AVS with ACTH (successful/unsuccessful))	26 (57.8%)/19 (42.2%)	10 (38.5%)/13 (61.5%)	*p* = 0.264

**Table 4:** Adrenal venous sampling results in the whole cohort and in the different DST categories (Corrected aldosterone: aldosterone/cortisol ratio. DST: dexamethasone suppression test; ACTH: adrenocorticotropic hormone; CSI: contralateral suppression index; AVS: Adrenal venous sampling).

	Whole Cohort (n = 225)	DST ≤ 1.8 µg/dL (n = 127)	DST > 1.8 µg/dL (n = 98)	*p* Value
Cortisol dominant vein (µg/dL)				
Basal	76 (32.3–315.6)	82.2 (31.7–438.6)	73.9 (33.8–254.6)	*p* = 0.942
Post ACTH	634 (239–1166)	634 (222–1206)	634 (269–897.3)	*p* = 0.907
Cortisol non-dominant vein (µg/dL)				
Basal	171 (35.6–420.9)	132.3 (28.2–528)	205.4 (38.9–378.2)	*p* = 0.366
Post ACTH	621.6 (57.7–1054)	627.8 (103–1106)	331.1 (41.3–878)	*p* = 0.628
Corrected aldosterone dominant vein (ng/µg)				
Basal	3.54 (1.37–13.86)	4.1 (1.49–11.52)	2.93 (1.3–14.32)	*p* = 0.737
Post ACTH	6.66 (2.52–19.44)	5.9 (2.4–21.04)	6.73 (3.21–15.85)	*p* = 0.644
Corrected aldosterone non-dominant vein				
Basal	0.70 (0.32–1.50)	0.73 (0.4–1.84)	0.69 (0.3–1.28)	*p* = 0.223
Post ACTH	1.42 (0.59–5.42)	1.35 (0.5–6.24)	1.58 (0.71–4.78)	*p* = 0.741
Selectivity index dominant vein				
Basal	7.68 (3.55–28)	9 (3.61–38)	6.88(3.54–22.92)	*p* = 0.439
Post ACTH	13.19 (6–26.9)	12.51 (6.23–31.5)	13.2 (2.9–15.08)	*p* = 0.277
Selectivity index non-dominant vein				
Basal	13.8 (3.91–34.02)	13.51 (3.04–35.94)	13.8 (5.34–28.36)	*p* = 0.731
Post ACTH	13.28 (1.65–25.46)	14 (1.6–30.7)	8.8 (1.8–14.7)	*p* = 0.552
Lateralization index (all)	4.43 (1.88–13.5)	3.69 (1.78–12.34)	4.68(2.04–14.78)	*p* = 0.318
Basal	4.48 (1.81–15.4)	4.41 (1.72–13.52)	4.724 (2.07–19.82)	*p* = 0.575
Post ACTH	3.8 (1.95–6.35)	3.36 (1.94–5.29)	4.65 (2–12.8)	*p* = 0.352
CSI (all)	0.47 (0.19–0.98)	0.48 (0.21–1.12)	0.43 (0.19–0.79)	*p* = 0.791
Basal	0.44 (0.18–0.93)	0.47 (0.17–1.18)	0.4 (0.19–0.76)	*p* = 0.716
Post ACTH	0.66 (0.32–1.06)	0.55 (0.3–1.03)	0.81 (0.66–2.29)	*p* = 0.357
AVS results:				*p* = 0.277
Unilateral	47.1%	44.1%	51.0%	
Bilateral	35.1%	34.6%	35.7%	
Unsuccessful	17.8%	21.3%	13.3%	

## Data Availability

The datasets generated and analysed during the current study are available from the corresponding author on reasonable request.
